# War-related continuous traumatic stress as a potential mediator of associations between moral distress and professional quality of life in nurses: a cross-sectional study in Ukraine

**DOI:** 10.1186/s12912-024-02668-4

**Published:** 2025-01-06

**Authors:** Larysa Zasiekina, Anastasiia Martyniuk

**Affiliations:** 1 Department of Psychology, University of Exeter, Exeter, UK; 2 Department of General and Clinical Psychology, Lesya Ukrainka Volyn National University, Lutsk, Ukraine

**Keywords:** Moral distress, War-related continuous traumatic stress, Compassion fatigue, Burnout, Secondary traumatic stress, Compassion satisfaction, Nurses

## Abstract

**Background:**

At the beginning of 2022, Central Europe entered a state of emergency due to the Russian invasion of Ukraine. Nurses were particularly vulnerable to a decline in their professional quality of life, facing repeated exposure to military trauma, ethical dilemmas, prolonged working hours, and increased stress and fatigue. This study aimed to contribute to our understanding of the potential mediating effect of war-related continuous traumatic stress on the association between moral distress and professional quality of life, including compassion satisfaction and compassion fatigue, represented by burnout and secondary traumatic stress.

**Methods:**

This study used the Professional Quality of Life (ProQOL) Scale to assess compassion fatigue, including burnout, secondary traumatic stress, and compassion satisfaction; the Moral Distress Questionnaire for Nurses to examine everyday moral distress in healthcare facilities and everyday ethical dilemmas of nurses; and the Continuous Traumatic Stress Response (CTSR) Scale to assess ongoing threats, resulting in exhaustion/rage, and fear/betrayal. The sample consisted of 130 female nurses (mean age 40.20 ± 12.15 years) from general surgery, neurosurgery, trauma and orthopaedic surgery, and urgent care who had been undergoing advanced training to work with injured military personnel at the Volyn Medical Institute (Ukraine) from March to May 2023. All nurses had experienced direct and indirect exposure to military trauma, and 105 participants were working with injured military personnel.

**Results:**

The results of the mediation analysis indicated that war-related continuous traumatic stress fully mediated the relationship between moral distress and different components of professional quality of life, namely, compassion fatigue, including burnout and secondary traumatic stress, and compassion satisfaction.

**Conclusion:**

Research has shown that continuous traumatic stress has a significant effect on the relationship between moral distress and various aspects of professional quality of life, underscoring the need for targeted interventions for nurses facing war-related trauma.

## Background

At the beginning of 2022, a state of emergency was declared in Central Europe due to the Russian invasion of Ukraine. Nurses found themselves at a high risk for poor professional quality of life, experiencing multiple exposures to military trauma, ethical challenges, extended working hours, and heightened levels of stress and fatigue. Recent research indicates that risk factors for nurses’ well-being including high workload, emotional demands, staffing shortages, irregular hours, lack of resources, administrative demands, and patient complexity, intensify during wars, epidemics, and pandemics [1].

The ethical challenges in nursing practice within a high-risk environment have revealed a phenomenon widely described in the pandemic literature – moral distress. Moral distress in healthcare professionals encompasses emotional suffering after confronting situations that violate professional ethics. In the context of the COVID-19 pandemic, nurses are involved in complex decision-making and moral dilemmas related to satisfying patients’ needs with limited resources [2–7].

In Ukraine, the initial phase of the pandemic was marked by inadequate testing systems, overwhelming workloads, and limited opportunities for nurses to maintain contact with their families. These factors significantly increased the emotional burden on healthcare personnel [8]. Over time, the emotional exhaustion and moral distress experienced during the pandemic evolved into continuous traumatic stress with the full-scale Russian invasion of Ukraine. This transformation highlights the compounded psychological and ethical challenges faced by Ukrainian nurses in navigating ongoing threats [9].

A review of the literature revealed recent studies highlighting the distressing psychological effects of wars and armed conflicts on the mental health of nurses delivering care in the war zone hospitals [10–13]. However, these papers primarily present the results of qualitative studies on the negative impact of war-related circumstances on the health of military nurses, highlighting a gap in quantitative research that could deepen our understanding of the effects of war-related trauma on nurses.

A recent systematic literature review highlighted higher rates of moral distress in military healthcare personnel compared to civilians, manifesting through physiological, psychological, social, or spiritual symptoms [14]. However, the review’s samples for moral distress studies included significantly fewer military settings compared to civilian medical centers (four and ninety-six, respectively). Furthermore, the review paid minimal attention to the role of nurses as a specific professional group of clinical healthcare personnel exposed to both direct and indirect trauma in the shared traumatic reality of the rear, where they provide care to both service members and civilians.

Despite increased attention given to moral distress in the nursing workforce during the COVID-19 pandemic, there is limited evidence available on the association between moral distress and professional quality of life, specifically in terms of compassion fatigue and compassion satisfaction, under war-related continuous traumatic stress [15]. The professional quality of life of caring professionals, introduced by Stemm [16] and then revised by Heritage et al. [17], includes compassion satisfaction, which is the pleasure derived from helping others, and compassion fatigue, which is represented by burnout and secondary traumatic stress arising from chronic workplace stress. Existing studies suggest a medium to strong association between moral distress and compassion fatigue, particularly in nursing personnel [18–22]. Age, sex, marital status and education are the main demographic characteristics associated with professional quality of life. However, there is little agreement on demographic characteristics as factors for developing compassion fatigue and compassion satisfaction in nurses working in high-risk environments [23–25]. Therefore, despite the acknowledged moral distress as a risk factor for the professional quality of life in the nursing workforce, there remains a scarcity of evidence regarding the association between moral distress and compassion fatigue and compassion satisfaction under war-related continuous traumatic stress. Continuous traumatic stress refers to the impact of prolonged and persistent exposure to threats, distinguishing between ongoing and finite experiences. This concept places emphasis on the current and potential effects of trauma instead of on past traumatic events, as has been done in the case of posttraumatic stress disorder (PTSD) [26; 27]. Additionally, continuous traumatic stress differs from complex PTSD by focusing on collective violence, such as wars, terrorist attacks, and armed conflicts, rather than interpersonal trauma [28].

**Fig. 1:**
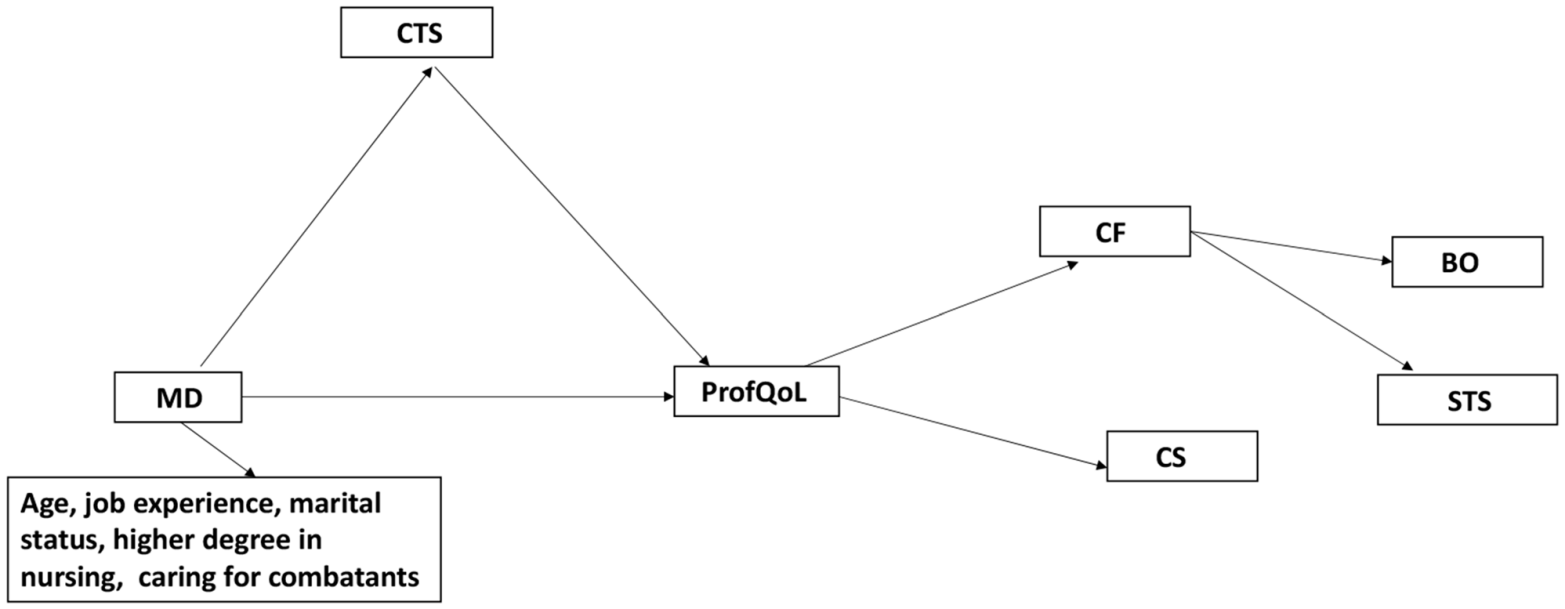
Mediating effect of war-related continuous traumatic stress on moral distress and professional quality of life. Note. CTS = continuous traumatic stress; MD = moral distress; ProfQol = professional quality of life; CF = compassion fatigue, CS = compassion satisfaction; BO = burnout; STS = secondary traumatic stress

This study aimed to contribute to our understanding of the potential mediating effect of war-related continuous traumatic stress on the association between moral distress and professional quality of life, including compassion satisfaction, compassion fatigue, represented by burnout and secondary traumatic stress in nurses (see Fig. 1).

## Methods

### Research design

The cross-sectional exploratory survey was conducted between March and May 2023 at the Volyn Medical Institute, where nurses were receiving advanced training to care for injured military personnel. While the nurses who participated in our study do not work on the frontline and provide care to the wounded in the relatively calm western part of Ukraine, according to the International Rescue Committee (2024), one in five surveyed healthcare workers in Ukraine experience prolonged war-related stress, including anxiety, uncertainty, and a decline in life satisfaction and self-worth while delivering care to war-affected people across all oblasts of Ukraine. Volyn Oblast [administrative unit], located far from the frontlines of the Russia-Ukraine war, has become a key region for the treatment and rehabilitation of injured combatants and civilians from the eastern and southern areas of the country, with nurses’ activities marked by heightened stress, long working hours, and shortages of supplies.

### Participants and settings

A power analysis was not performed because we did not know the effect size of the mediation analyses. The administration of the Volyn Medical Institute supported the recruitment of participants. In total, 137 nurses completed the survey; however, nurses from intensive care and oncology departments were excluded from the sample because they experienced significantly greater compassion fatigue than nurses in other hospital units due to the specifics of their work [29]. The final sample consisted of 130 female nurses from general surgery, neurosurgery, trauma and orthopaedic surgery, and urgent care. All nurses had experienced direct and indirect exposure to war-related trauma. Online consent forms and questionnaires were shared with the nurses to participate in the survey.

### Measures

The ProQOL Scale is a 30-item self‐report measure used to assess well-being and attitudes toward work during the last month. Each item on these measures is rated on a 5-point Likert scale (from 1 to 5) according to the frequency with which certain situations or experiences were encountered in the last 30 days [16]. The scale consists of three subscales: compassion satisfaction, burnout, and secondary traumatic stress. Burnout and secondary traumatic stress together make up the compassion fatigue subscale. Burnout is associated with feelings of hopelessness and difficulty performing effectively. The internal consistency of burnout was α = 0.75. Secondary traumatic stress refers to the secondary impact of extremely or traumatic stressful events on work (α = 0.81). Compassion satisfaction is the satisfaction a nurse derives from being able to do their job well (α = 0.88). The maximum score for each subscale is 50. A raw score of 22 or less indicates a low level, a score of 23 to 41 indicates a moderate level, and a score of 42 or more indicates a high level.

The Moral Distress Questionnaire for Nurses is designed to measure everyday moral distress in healthcare facilities and focuses on everyday ethical dilemmas of healthcare workers [30]. The questionnaire consists of 9 questions and contains two dimensions: the level of moral distress (first six questions, α = 0.78) and tolerance of moral dilemmas (last 3 questions, α = 0.62). Items were rated on a 4-point Likert scale (from 1 to 4) according to agreement with statements. Total scores range from 0 to 36, with higher scores indicating greater moral distress. Compared with other moral distress scales, this questionnaire focuses primarily on everyday ethical dilemmas and can be used in different healthcare settings.

The Continuous Traumatic Stress Response (CTSR) Scale is designed to measure people’s well-being over the past month [26]. The scale consists of 11 items, each rated on a 4-point scale ranging from 0 = not at all to 3 = extremely, and summed for a continuous traumatic stress score. The original scale consists of 3 subscales: exhaustion/detachment (α = 0.86), rage/betrayal (α = 0.82), and fear/helplessness (α = 0.74). Total scores range from 0 to 33, with higher scores indicating higher levels of continuous traumatic stress. The internal consistency of the total scale is 0.90. The Ukrainian version of the CTSR Scale, which was cross-culturally adapted and validated by Zasiekina and colleagues, demonstrates high internal consistency (α = 0.86) and includes two subscales, “Exhaustion and Rage” and “Fear and Betrayal” [28].

Participant demographic data are presented in Table 1.

The mean age of the nurses was 40.20 ± 12.15 years, with a mean work experience of 19.65 ± 12.86 years. Most of the participants were married (70.80%) and had experience caring for combatants (80.8%). About one-fifth of the participants (17.70%) had a higher education degree in nursing. In terms of trauma, more than half of the participants (53.10%) experienced direct trauma, particularly life-threatening situations involving missile explosions, and 46.90% experienced indirect trauma, such as witnessing trauma to others and observing the demolition of property.

**Table 1:** Demographic data for the nurses from general surgery, neurosurgery, trauma and orthopaedic surgery, and urgent care (*n* = 130)

	*n*		%					
Marital status Single/Married	38/92		29.20/70.80					
Higher education degree in nursing Yes/No	23/107		17.70/82.30					
Caring for combatants Yes/No	105/23		80.80/19.20					
				Min	Max	Mean	SD	
Age		130		20	74	40.20	12.15	
Work experience		130		1	53	19.65	12.86	

The data were analysed using IBM SPSS statistical software version 29.0. The mediating role of continuous traumatic stress in the relationship between moral distress and professional quality of life was examined using the PROCESS v3 macro in SPSS with 5,000 bootstrapped samples [31]. Three separate mediation models were estimated with the ProQoL subscales (burnout, secondary traumatic stress and compassion satisfaction) as the outcome variables. In these models, the indirect effect was considered statistically significant if the 95% bias-corrected confidence interval did not contain zero. We examined a number of variables as potential confounders, as they have been shown to be associated with moral distress, compassion fatigue and compassion satisfaction in other studies and could confound the relationships of interest in our study [32]. These included age, work experience, marital status, degree of nursing and caring for combatants. As all potential confounders did not change the unadjusted odds ratios by more than 5%, we excluded them from the final model.

## Results

Moral distress in nurses did not differ according to demographic variables, particularly marital status (*t*(128) = -1.18, *p* = .24, 95% CI for [-2.45, 0.62], *d* = − 0.23) or a higher degree of nursing (*t*(128) = 0.45, *p* = .33, 95% CI [-1.43, 45, 2.25. ], *d* = 0.10); and caring for combatants (*t*(128) = 0.12, *p* = .45, 95% CI [-1.68, 1.88.], *d* = 0.03). Moral distress and age and moral distress and work experience were not found to be correlated, *r*(128) = 0.07, *p* = .41; and *r*(128) = 0.06, *p* = .53, respectively.

According to Stamm’s [16] interpretation, 62% of respondents who experienced a low level of burnout scored 22 or less, and 38% had a moderate level of burnout (see Table 2). There were no nurses with a high level of burnout. Fifty-five per cent of the nurses had a low level of secondary traumatic stress (22 or less), and 45% had a moderate level of secondary traumatic stress (23 to 41). Concerning burnout, no nurses with a high level of secondary traumatic stress scored 42 or higher. Forty-nine per cent of the nurses had a high level of compassion satisfaction (42 or higher), 50% had an average level of compassion satisfaction (23 to 41), and 1% had a low level of compassion satisfaction (22 or less).

**Table 2:** Average scores of nurses on burnout, secondary traumatic stress, compassion satisfaction, moral distress and continuous traumatic stress (*n* = 130)

Scale	Min–Max possible values	Min–Max values taken from the scale	Mean (SD)
Burnout	0–50	10–38	21.12 (5.98)
Secondary traumatic stress	0–50	10–38	22.59 (5.43)
Compassion satisfaction	0–50	21–50	41.47 (5.77)
Moral distress	0–36	9–28	15.52 (4.03)
Continuous traumatic stress	0–33	0–22	4.60 (4.45)

The results of the mediation analysis are presented in Table 3.

Since burnout and secondary traumatic stress together make up the compassion fatigue subscale, the study applied models predicting moral distress and burnout and secondary traumatic stress as components of compassion fatigue through war-related continuous traumatic stress.

In the model predicting moral distress and burnout, moral distress predicted war-related continuous traumatic stress (Path a; b = 0.40, 95% CI [0.215, 0.575], *p* < .001), and war-related continuous traumatic stress predicted burnout (path b; b = 7.07, 95% CI [5.040, 9.107], *p* < .001). The total effect of war-related continuous traumatic stress on moral distress and burnout was significant (b = 4.90, 95% CI [2.461, 7.344], *p* < .001). This might be explained by the fact that nurses experiencing continuous traumatic stress during the first half of the second year of the full-scale invasion of Ukraine had greater symptoms of moral distress and burnout than did nurses working in peaceful times. The indirect effect of war-related continuous traumatic stress on moral distress and burnout was significant, suggesting that war-related continuous traumatic stress mediated this association (b = 2.795, 95% CI [1.113, 4.722]). This significant mediation effect represented 57% of the total effect. The direct effect became nonsignificant after controlling for war-related continuous traumatic stress in the mediation model (b = 2.108, 95% CI [-0.133, 4.349], *p* = .650), suggesting that war-related continuous traumatic stress fully mediated the relationship between moral distress and burnout.

In the model predicting moral distress and secondary traumatic stress, moral distress predicted war-related continuous traumatic stress (Path a; b = 0.40, 95% CI [0.215, 0.575], *p* < .001), and war-related continuous traumatic stress predicted secondary traumatic stress (path b; b = 5.83, 95% CI [3.905, 7.764], *p* < .001). The total effect of war-related continuous traumatic stress on moral distress and secondary traumatic stress was significant, suggesting that nurses caring for injured combatants who often have PTSD symptoms might experience secondary traumatic stress (b = 4.272, 95% CI [2.035, 6.509], *p* < .001). The indirect effect of war-related continuous traumatic stress on moral distress and secondary traumatic stress was significant, suggesting that war-related continuous traumatic stress mediated this association (b = 2.305, 95% CI [0.831, 3.906]). This significant mediation effect represented 54% of the total effect. The direct effect became nonsignificant after controlling for war-related continuous traumatic stress in the mediation model (b = 1.967, 95% CI [-0.151, 4.092], *p* = .070), suggesting that war-related continuous traumatic stress fully mediated the relationship between moral distress and secondary traumatic stress.

In the model predicting moral distress and compassion satisfaction, moral distress predicted war-related continuous traumatic stress (Path a; b = 0.395, 95% CI [0.215, 0.575], *p* < .001), and war-related continuous traumatic stress predicted compassion satisfaction (path b; b = -4.894, 95% CI [-7.120, -2.668], *p* < .001). The total effect of war-related continuous traumatic stress on moral distress and compassion satisfaction was significantly negative, suggesting that continuous traumatic stress serves as a significant barrier to compassion satisfaction, a key component of professional quality of life in nurses (b = -2.464, 95% CI [-4.908, − 0.021], *p* = .048). The indirect effect of war-related continuous traumatic stress on moral distress and compassion satisfaction was significant, suggesting that war-related continuous traumatic stress mediated this association (b = -1.934, 95% CI [-3.894, − 0.539]). This significant mediation effect represented 78% of the total effect. The direct effect became nonsignificant after controlling for war-related continuous traumatic stress in the mediation model (b = − 0.531, 95% CI [-2.983, 1.214], *p* = .669), suggesting that war-related continuous traumatic stress fully mediated the relation between moral distress and compassion satisfaction.

**Table 3:** Summary of mediation results (*n* = 130)

Outcome	CTS-ProQoL Controlling for MD	MD- ProQoL	Mediation: MD-CTS-ProQoL	
	b Path	Total effect (c path)	Indirect Path (ab Path)	Direct Path (c’ Path)
	B_b_ (95%) CI; β_b_	*b*_c_ (95% CI); β_c_	(*b*_a_ x *b*_b_) (95% CI); β_ab_	*b*_c’_ (95% CI); β_c’_
BO	7.07*** (5.04; 9.11), 1.03	4.90***(2,46; 7.34),1.23	2.80 (1.11; 4.72), 0.91	2.11 (-0.13; 4.35), 1.13
STS	5.83*** (3.91, 7.76), 0.98	4.27***, (2.04, 6.51), 1.13	1.93, (-3.89, − 0.54), 0.77.	− 0.53, (-2.98, 1.21), 0.1.07
CS	-4.89***, 95% CI (-7.12, -2.67), 1.12	-2.46*** (-4.91, − 0.02), 1.24	-1.93***, 95% CI (-3.89, − 0.54), 0.86	− 0.53, 95% CI (-2.98, 1.21), 1.24

Note. CTS = continuous traumatic stress; ProQoL = professional quality of life; BO = burnout; STS = secondary traumatic stress; CS = compassion satisfaction, *b* represents unstandardised regression weights, and β represents partially standardised regression weights

***p < .001

## Discussion

To our knowledge, this is the first cross-sectional study to explore the role of continuous traumatic stress as a mediator of the relationship between moral distress and compassion fatigue, represented by burnout and secondary traumatic stress, and compassion satisfaction in nurses in the shared reality of war. The findings show no differences in moral distress according to demographic variables, particularly marital status, higher degree of nursing, caring for combatants, age and work experience. Most nurses demonstrated low and moderate levels of burnout and secondary traumatic stress, and most of them had a high and average level of compassion satisfaction. The results of the mediation analysis indicated that war-related continuous traumatic stress fully mediated the relationship between moral distress and different components of professional quality of life, namely, compassion fatigue, including burnout and secondary traumatic stress, and compassion satisfaction.

The results of this study showed that less than half of the nurses experienced moderate levels of burnout and secondary traumatic stress. However, a greater percentage of nurses experienced a moderate level of secondary traumatic stress than did nurses who experienced burnout. This result is in line with a previous systematic review indicating a greater prevalence of secondary traumatic stress in nurses working with people who experienced trauma [30]. A possible explanation for this might be that their experience included not only physical injuries but also invisible wounds. Nurses might be exposed to repeated war-related stories of combatants and civilians during their close relationships with patients.

Additionally, nurses caring for injured individuals in surgical divisions are regularly exposed to trauma, suffering, and death. Witnessing these severe cases in a shared traumatic reality where nurses experience their own war-related traumas might lead to negative personal and professional outcomes [33, 34]. Further studies with a deeper focus on secondary traumatic stress and burnout in nurses caring for injured combatants and civilians during wars are therefore suggested.

One unanticipated result was that almost half of the nurses had high levels of compassion satisfaction. These results are in line with other studies suggesting that nurses can experience compassion satisfaction with critically ill patients and traumatised military service [35]. The main predictors of compassion satisfaction in healthcare staff caring for patients in critical condition are nursing competence, compassion competence and efficient communication, workload reduction, marital status and educational promotion [36, 37]. The results of our research do not indicate that marital status and a higher degree of nursing education are potential confounders in models predicting moral distress, secondary traumatic stress, burnout, and compassion satisfaction. Additionally, gender and age are not related to moral distress in nurses who experienced direct and indirect exposure to war-related trauma. These results are not in line with recent research indicating age and gender as significant predictors of moral distress in this professional group [38]. However, the authors of a recent study focused on perceived organisational support as a protective factor against moral distress in nurses during the COVID-19 pandemic, which might have a different effect than war-related continuous traumatic stress. Another possible explanation might be that moral distress is more closely related to the nature of the ethical dilemmas and the context in which care is provided rather than personal or demographic characteristics. The intense and often traumatic nature of wartime healthcare may create war-related moral distress across different groups of nurses. These results align with the findings of a recent systematic review in which moral distress in military critical care nurses arises from inconsistency between their personal and professional beliefs, leading to a loss of moral integrity in everyday settings [38]. Klimentidou et al. noted that a professional rank has an effect on the compassion satisfaction of military nurses, with lower compassion satisfaction for head nurses than for nurse managers [39]. Further studies that take age, gender, marital status, degree in nursing, work experience and types of war-related trauma into account in assessing moral distress and its relationship with professional quality of life had to be undertaken.

Additional research is needed to better understand whether nurses may experience compassion satisfaction resulting from their professional contributions to society during the war period. This exploration could shed light on how nurses’ perceptions of their roles and accomplishments decrease their moral distress and positively impact their professional quality of life. Further study of how professional commitments might gain a sense of fulfilment and compassion satisfaction in nurses caring for patients with war-related injuries is needed.

The findings indicate that participants did not demonstrate a high level of war-related continuous traumatic stress in terms of exhaustion and rage or fear and betrayal. These results may be due to nurses’ adjustment to life-threatening situations over a year. In this context, we could speak not only about war-related “wear and tear” effects on mental health but also about “wear and bear” effects, such as increasing resilience in repeated traumatic situations [26].

Importantly, war-related continuous traumatic stress fully mediated the relationship between moral distress and compassion fatigue, represented by burnout and secondary traumatic stress, as well as between moral distress and compassion satisfaction. There are several possible explanations for this result. First, nurses in our sample worked in surgical divisions, which are not at high risk for moral distress and compassion fatigue compared to healthcare staff from intensive care, emergency, oncology and psychiatric hospitals [29, 40]. Therefore, the significant interaction between moral distress and compassion fatigue might be affected by individuals’ own experience of war-related continuous traumatic stress. Additionally, the number of injured patients and the severity of their trauma are intensively increasing during the ongoing war. Second, in healthcare settings in Ukraine, a post-Soviet state, the treatment plans and life-preserving interventions in surgical departments are primarily conducted by physicians rather than nurses. Physicians are primarily responsible for creating effective treatment plans and making critical decisions, unlike practices in many developed countries where nurses play a more significant role. Therefore, moral distress and compassion fatigue in nurses in surgical divisions might be affected more by experiencing war-related stress than by experiencing everyday ethical dilemmas of decision-making. Finally, burnout and secondary traumatic stress, as elements of compassion fatigue, might be associated with moral pain under the accumulated effect of one’s own experience of war-related trauma and patients’ narratives on life-threatening situations in war [41]. Further study is needed to develop a complete picture of the effects of direct and indirect war-related trauma on the professional quality of life. Future research should also explore other factors and mechanisms contributing to moral distress, aiming to develop targeted interventions to support nurses in high-risk environments.

### Study limitations and future directions

This study had several limitations that should be addressed. It relied on a cross-sectional survey and, therefore, cannot make any conclusions regarding causality. Second, it was conducted during the second year of the Russian-Ukrainian War and cannot evaluate potential long-term consequences. Additionally, there were no prewar baseline data on moral distress and professional quality of life before the full-scale invasion of Ukraine. A longitudinal study could show whether moral distress fades or worsens over time under the circumstances of continuous traumatic stress. The survey was conducted online, and the participants’ responses might differ from those in a face-to-face assessment.

## Conclusion

The current study examined continuous traumatic stress as a mediator of the relationship between moral distress and compassion fatigue (represented by burnout and secondary traumatic stress) and compassion satisfaction in nurses within the shared traumatic reality of the full-scale invasion of Ukraine. The study showed that war-related continuous traumatic stress fully mediated the relationship between moral distress and compassion fatigue, as well as between moral distress and compassion satisfaction. The results of the study have profound theoretical implications for the nursing profession, particularly in the high-risk environment of multiple exposures to war-related trauma and address the unique ethical challenges faced by nurses in such settings. Ensuring that nurses receive adequate training and resources to cope with continuous traumatic stress is crucial for maintaining their professional quality of life and the quality of patient care. It is recommended that resilience-building and stress-management interventions target war-related continuous traumatic stress associated with moral distress in nurses caring for both civilian and military populations.

## Data Availability

Raw data that support the findings of the study have been deposited in Mendeley Data.Zasiekina, Larysa (2024), “War-related continuous traumatic stress as a potential mediator of associations between moral distress and professional quality of life in nurses: a cross-sectional study in Ukraine”, Mendeley Data, V1, 10.17632/t4wtnh4zpg.1.
